# Global gene expression of the inner cell mass and trophectoderm of the bovine blastocyst

**DOI:** 10.1186/1471-213X-12-33

**Published:** 2012-11-06

**Authors:** Manabu Ozawa, Miki Sakatani, JiQiang Yao, Savita Shanker, Fahong Yu, Rui Yamashita, Shunichi Wakabayashi, Kenta Nakai, Kyle B Dobbs, Mateus José Sudano, William G Farmerie, Peter J Hansen

**Affiliations:** 1 Department of Animal Sciences and D.H. Barron Reproductive and Perinatal Biology Research Program, PO Box 110910, Gainesville, FL, 32611-0910, USA; 2 Interdisciplinary Center for Biotechnology Research, University of Florida, Gainesville, FL, 32611-0910, USA; 3 Kyushu-Okinawa Agricultural Research Center, National Agriculture and Food Research Organization, Kumamoto, Japan; 4 Laboratory of Developmental Genetics, Institute of Medical Science, University of Tokyo, Tokyo, Japan; 5 Human Genome Center, Institute of Medical Science, University of Tokyo, Tokyo, Japan; 6 Departamento de Reprodução Animal e Radiologia Veterinária, Faculdade de Medicina Veterinária e Zootecnia, UNESP, Botucatu, São Paulo, Brasil

**Keywords:** Blastocyst, Trophectoderm, Inner cell mass, Development

## Abstract

**Background:**

The first distinct differentiation event in mammals occurs at the blastocyst stage when totipotent blastomeres differentiate into either pluripotent inner cell mass (ICM) or multipotent trophectoderm (TE). Here we determined, for the first time, global gene expression patterns in the ICM and TE isolated from bovine blastocysts. The ICM and TE were isolated from blastocysts harvested at day 8 after insemination by magnetic activated cell sorting, and cDNA sequenced using the SOLiD 4.0 system.

**Results:**

A total of 870 genes were differentially expressed between ICM and TE. Several genes characteristic of ICM (for example, *NANOG*, *SOX2*, and *STAT3*) and TE (*ELF5*, *GATA3*, and *KRT18*) in mouse and human showed similar patterns in bovine. Other genes, however, showed differences in expression between ICM and TE that deviates from the expected based on mouse and human.

**Conclusion:**

Analysis of gene expression indicated that differentiation of blastomeres of the morula-stage embryo into the ICM and TE of the blastocyst is accompanied by differences between the two cell lineages in expression of genes controlling metabolic processes, endocytosis, hatching from the zona pellucida, paracrine and endocrine signaling with the mother, and genes supporting the changes in cellular architecture, stemness, and hematopoiesis necessary for development of the trophoblast.

## Background

Following its formation by syngamy of the pronuclei of the oocyte and sperm, the mammalian embryo begins life as a totipotent, single cell organism. Subsequent cycles of cell division and the formation of tight junctions between blastomeres lead to a condition whereby blastomeres on the outer face of the embryo exhibit different patterns of cell polarity, gene expression and protein accumulation than blastomeres on the inner part of the embryo [1-4]. Non-polarized blastomeres in the inner part of the embryo are destined to form the pluripotent inner cell mass (ICM) that gives rise to the embryo while polarized cells in the outer face of the embryo are fated to differentiate into the trophectoderm (TE), which develops into extraembryonic membranes. Cell fate may be determined as early as the 4–8 cell stage in the mouse and depend upon differences between blastomeres in the kinetics of the interaction between the transcription factor Pou5f1 and DNA binding sites [5]. Nonetheless, blastomeres do not undergo lineage commitment until about the 32-cell stage (in mice), based on loss of ability of blastomeres to form either ICM or TE [2].

Lineage commitment towards ICM or TE is under the control of specific transcription factors. The exact role of at least some transcription factors varies with species [6]. In the best studied species, the mouse, the ICM is regulated by *Sall4*, *Pou5f1*, *Sox2* and *Nanog* while TE formation results from a cascade of events involving *Yap1*, *Tead4*, *Gata3*, *Cdx2*, *Eomes* and *Elf5*[7]. Functional properties of the two cell lineages is also divergent. In part, this reflects the processes responsible for establishment and maintenance of cell lineage, such as differences in transcription factor usage, cell signaling pathways and epigenetic marks [7,8]. In addition, the function of the ICM, which is fated to undergo a series of differentiation events in the gastrulation process, is different from the TE, which is destined to interact with the lining of the maternal reproductive tract.

In the present study, we describe, for the first time, differences in the transcriptome of the ICM and TE with the objective of understanding the consequences of the differentiation of these two cell types for cellular function. This was achieved by separating ICM and TE using a newly-developed immunomagnetic procedure [9] followed by next-generation sequencing. Results reveal the implications of the spatial and developmental differentiation of these first two lineages of the preimplantation embryo with respect to metabolism, interaction with the maternal system and changes in cellular architecture. In addition, aspects of molecular control of the process of lineage commitment and differentiation are illustrative of similarities and differences with the prototypical mouse model.

## Methods

### Reagents

All reagents were purchased from Sigma-Aldrich (St. Louis, MO, USA) or Fisher Scientific (Pittsburgh, PA, USA) unless otherwise specified.

### Embryo culture and ICM/TE isolation

Bovine embryos were produced from slaughterhouse-derived oocytes using procedures for in vitro oocyte maturation, fertilization, and embryo culture as described previously [10]. Ovaries were donated by Central Packing, Center Hill Florida. The day of fertilization was defined as Day 0. After fertilization for 18–20 h, embryos were cultured in SOF-BE1 medium [11] at 38.5°C in a humidified atmosphere of 5% CO_2_ and 5% O_2_ with the balance N_2_. Embryos were cultured in groups of 30 in a 50 μl culture drop under mineral oil. At Day 6, an additional 5 μl culture medium was added. At Day 8, blastocysts were harvested and used to prepare preparations of ICM and TE using magnetic activated cell sorting as reported previously [9].

Three separate pools of TE and ICM for each treatment were obtained. Each pool was prepared using 88 to 102 blastocysts. A total of 15 fertilization procedures were used to prepare the blastocysts; a set of three bulls was used for fertilization for each procedure.

### RNA preparation, library construction and sequencing using SOLiD 4 system

Total RNA was isolated from each pool of embryonic cells using the PicoPure RNA Isolation Kit (Applied Biosystems, Foster City, CA, USA) according to the manufacturer’s instructions. The quality of RNA was assessed using the Agilent 2100 Bioanalyzer (Agilent Technologies, Santa Clara, CA). Amplified cDNA was prepared from total RNA for RNA-Seq applications using the Ovation RNA-Seq kit (NuGen Technology, San Carlos, CA). Barcoded fragment libraries were constructed using the SOLiD^TM^v4 fragment library kit according to the manufacturer’s protocol (Applied Biosystems). Briefly, double stranded cDNA was sheared to 150–180 bp fragments using a Covaris^TM^S2 Sonication system (Covaris, Woburn, MA). The fragmented DNA was subsequently end-repaired and blunt-end ligated to P1 and P2 adaptors. The adaptor ligated, purified and size-selected 200–270 bp fragments were nick-translated and then amplified using primers specific to P1 and P2 adaptors and Platinum® PCR Amplification Mix (Applied Biosystems). The quality of the libraries and fragment distribution were verified by running 1 μl of each library on Agilent DNA 1000 chip (Agilent Technologies). Amplified libraries (5 different libraries pooled for each slide) were immobilized onto SOLiD P1 DNA beads (Applied Biosystems). The bead-bound libraries were then clonally amplified by emulsion PCR according to the Applied Biosystems SOLiD^TM^ 4 Systems Templated Bead Preparation Guide. After amplification, emulsions were disrupted with 2-butanol and the beads containing clonally amplified template DNA were P2-enriched and extended with a bead linker by terminal transferase. The quantity of the beads was determined using a NanoDrop® ND1000 spectrophotometer (Thermo Scientific, Wilmington, DE). Approximately 600-700M beads were deposited on each slide (ran in total three slides) and sequenced using ‘sequencing by ligation’ chemistry and the 50x5 bp protocol on the SOLiD^TM^ v4 sequencer (Applied Biosystems) at the Interdisciplinary Center for Biotechnology Research, University of Florida. Results were obtained as color space fasta files.

### Analysis of read data

Raw sequencing reads were initially processed with GenomeQuest tools [12]. Ambiguous residues were trimmed off from both sides of the sequence. Bases with Phred quality below 12 from the 3’ end of the sequence were removed. Reads that were shorter than 40 bases or that contained more than 10 bases with quality below 12 were also discarded as were reads consisting of repetitive single bases that accounts for more than 60% of the length at the 3’ end. About 53 ~ 64% of reads were retained after clean up, proving 102–157 million clean reads for the three replicates of each treatment.

For mapping to the genome, the *Bos taurus* genomic sequence *bosTau4* (repeat masked) was downloaded from the UCSC genome browser (http://genome.ucsc.edu/). Sequencing reads of each sample were mapped independently to the reference sequences using TopHat 1.2.0 [13]. TopHat split reads to segments and joins segment alignments. A maximum of one mismatch in each of the 25 bp segments was allowed. This step mapped 36.8% reads to the genome. The unmapped reads were collected and mapped to the reference using Bowtie 0.12.7 [14] allowing three mismatches. Unmapped reads were further mapped to cDNA sequences using bfast 0.6.4 [15] while allowing for three mismatches for each read. The cDNA sequences of *B*. *taurus* were downloaded from the National Center of Biotechnology Information. Scaffold and chromosome sequences were cleared and a total of 35,842 sequences were obtained (http://www.ncbi.nlm.nih.gov/nuccore/?term=txid9913[Organism:noexp). Bfast aligned 27.6% of the total reads to the cDNA sequences. Therefore, a total of 64.4% or 595 million reads were mapped successfully. Of the mapped reads, 89.8% are uniquely mapped to either the genome or cDNA sequences. Data were deposited in the DDBJ Sequence Read Archive at http://www.ddbj.nig.ac.jp/index-e.html (Submission DRA000504).

Digital gene expression was determined as follows. The number of mapped reads for each individual gene was counted using the HTSeq tool (http://www-huber.embl.de/users/anders/HTSeq/doc/overview.html) with intersection-nonempty mode. HTSeq takes two input files - bam or sam-format files of mapped reads and a gene model file. The Ensemble gene annotation file in GTF format was downloaded from the UCSC genome browser. The DESeq package [16] in R was used for digital gene expression analysis. DESeq uses the negative binomial distribution, with variance and mean linked by local regression, to model the null distribution of the count data. Significant up- and downregulated genes were selected using two cutoffs: an adjusted P value of 0.05 and a minimum fold-change of 1.5.

### Classification of differentially expressed genes into gene ontology (GO) classes

Differentially expressed genes were annotated by the Database for Annotation, Visualization and Integrated Discovery (DAVID; (DAVID Bioinformatics Resources 6.7, http://david.abcc.ncifcrf.gov/) [17]. Most genes were annotated using the bovine genome as a reference and additional genes were annotated by comparison to the human genome. The DAVID database was queried to identify GO classes enriched for upregulated and downregulated genes. Functions of differentially expressed genes were further annotated using Kyoto Encyclopedia of Genes and Genomes (KEGG, http://www.genome.jp/kegg/). Overview of the differentially regulated KEGG pathways were mapped on KEGG Pathway Map using iPath2.0 (http://pathways.embl.de/) [18].

To further analyze patterns of genes differentially regulated between ICM and TE, k-mean clustering was performed. The reads count data of the 870 significant genes for the ICM-control versus TE-control comparison were clustered using k-means strategy [19]. To estimate the premium cluster number, k-values from 3 to 100 were tested and the corresponding sum of squares error (SSE) [20] was calculated for each k value. SSE is defined as the sum of the squared distance between each member of a cluster and its cluster centroid. The SSE values dropped abruptly until k = 8 (results not shown). To balance the minimum number of SSE and the minimum number of clusters, k = 8 was selected as the premium parameter for clustering genes and a heatmap was generated using *heatmap*.*2* of R package.

### Enrichment analysis for transcription factor binding sites

For each differentially expressed gene, the candidate promoter region was defined as the span of nucleotides from 200 bp upstream and 50 bp downstream from the transcriptional start site identified in Ensembl. To detect putative transcription factor binding sites (TFBS) in each promoter, we followed the method of Wasserman and Sandelin [21]. Position-specific weight matrices were obtained from the JASPAR database [22]. The score was calculated by formula 1 in Additional File 1. We also calculated the ratio of the score to the maximum score by formula 2 (Additional File 1). Statistical significance of each TFBS was evaluated by calculating the hypergeometric distribution using formula 3 (Additional file 1). We performed the ‘match’ program with ‘minSUM’ and ‘minFP’ thresholds to detect TFBS [23]. Statistical significance of each detected TFBS was evaluated by the hypergeometric distribution as described above.

### Calculation of GC contents and detection of CpG islands

The method by Gardiner-Garden and Frommer [24] was used to identify CpG islands in the region encompassing the 100 nucleotides upstream and 100 nucleotides downstream from the start site. Transcriptional start sites for differentially expressed genes were obtained from UMD3.1 [25]. For the definition of CpG islands, The GC content was calculated as ([C]+[G])/200, where [N] denotes the number of nucleotides “N” within the 200 base window. The CpG score was calculated as [CG]/([C]*[G]*200). A gene was classified as CpG positive when its GC content in the region spanning the 100 nucleotides upstream and the 100 nucleotides downstream from the start site exceeds 0.5 and when the CpG score in the same region exceeds 0.6. Otherwise, a gene was classified as CpG negative. Chi-square analysis was used to determine whether the percent of genes classified as CpG positive differed between 1) genes overexpressed in ICM versus genes overexpressed in TE and 2) genes overexpressed in ICM or TE versus the reference population of 25118 genes in the bovine genome.

### Confirmation of differences in gene expression between ICM and TE by quantitative PCR

An experiment was performed to verify the effect of cell type (ICM vs TE) and CSF2 on relative mRNA abundance of the *GATA3*, *ELF5*, *CDX2*, *NANOG* and *SOX2*. Embryos were prepared as described previously and blastocysts were collected at Day 7. Pools of 25–34 blastocysts were submitted to magnetic-activated cell sorting [9]. A total of 6 biological replicates of ICM and TE were prepared. mRNA extraction was performed using the All Prep DNA/RNA mini Kit (Qiagen, Inc., Valencia, CA, USA) followed by DNase (Qiagen) treatment and reverse transcription (High Capacity cDNA Reverse Transcription Kit, Applied Biosystems, Foster City, CA). Transcript abundance for *GATA3*, *ELF5*, *CDX2*, *NANOG* and *SOX2* as well as housekeeping genes *GAPDH*, *SDHA* and *YWHAZ* were quantified by a Bio-Rad thermal cycler CFX96-Real-Time system (Bio-Rad, Hercules, CA, USA) using SsoFast EvaGreen Supermix reagent (Bio-Rad, Hercules, CA, USA). PCR conditions were as follows: 30 sec at 95°C followed by 40 cycles each of 5 sec at 95°C and 1 min at 60°C. Data were analyzed using the delta-delta cycle threshold (Ct) method. The reference gene was the geometric mean of the Ct values of *GAPDH*, *SDHA* and *YWHAZ*. Primers for *ELF5* were based on NM_001024569.1 and were designed using PrimerQuest from idtDNA (http://www.idtdna.com) software, Efficiency was 95% and identity of amplicons was verified by sequencing products. The primers were 5’ TGCCATTTCAACATCAGTGGCCTG 3’ and 5’ AAGGCCACCCTCAAAGACTATGCT 3’. Other primer pairs were published previously: *GATA3*[26], *CDX2* and *NANOG*[9], *SOX2*[27] and *GAPDH*, *SDHA* and *YWHAZ*[28].

Data were analyzed by least-squares analysis of variance using the General Linear Model (GLM) procedure of the Statistical Analysis System, version 9.2 (SAS Institute Inc, Cary, NC, USA) Sources of variation in the model included cell type (ICM and TE), replicate and the interaction; cell type was considered fixed and replicate was considered random. Logarithmic transformation was applied to *CDX2* data to improve normality. All data are reported as untransformed least-squares means.

## Results

### Differentially expressed genes

The lists of differentially expressed genes, determined using an adjusted P value of ≤0.05 and ≥ 1.5-fold difference as cut-offs, are presented in Additional file 2. There were a total of 870 genes that were differentially expressed between ICM and TE, with 411 genes upregulated in the ICM and 459 downregulated in the ICM (i.e., upregulated in the TE).

### Annotation of genes differentially expressed between ICM and TE

Differentially expressed genes were annotated using the Gene ID conversion tool of the DAVID Bioinformatics Resources 6.7 (http://david.abcc.ncifcrf.gov/conversion.jsp); 835 of the 870 differentially expressed genes were annotated (389 genes upregulated in the ICM and 424 genes upregulated in the TE). For the list of genes upregulated in ICM, 10 GO terms were listed in the Biological Process group, 4 GO terms in the Cell Component group, and 5 terms in the Molecular Function group (Table 1). Terms related to transcriptional activities were dominant including regulation of transcription, DNA-dependent (25 genes), regulation of transcription from RNA polymerase II promoter (11 genes), DNA binding (29 genes), transcription regulator activity (22 genes) and transcription factor activity (17 genes). There were also GO terms related to metabolic activity including regulation of RNA metabolic process (25 genes), positive regulation of macromolecule metabolic process (12 genes), negative regulation of macromolecule metabolic process (10 genes), and enzyme binding (10 genes).

**Table 1:** **GO terms enriched for genes upregulated in the ICM as compared to TE**^**a**^

**GO term**	**Count**	**Percent**	**P value**	**FDR**^**b**^
**Biological Process**				
Regulation of transcription, DNA-dependent	25	6.7	0.04	43.9
Regulation of RNA metabolic process	25	6.7	0.04	49.9
Neurological system process	12	3.2	0.01	16.9
Regulation of cell proliferation	12	3.2	0.02	24.9
Immune response	12	3.2	0.03	35.4
Positive regulation of macromolecule metabolic process	12	3.2	0.03	43.1
Cognition	11	2.9	0.00	2.4
Regulation of transcription from RNA polymerase II promoter	11	2.9	0.02	29.6
Response to organic substance	10	2.7	0.01	16.8
Negative regulation of macromolecule metabolic process	10	2.7	0.04	43.9
**Cell Component**				
Plasma membrane	34	9.1	0.02	20.7
Extracellular region	30	8.0	0.00	4.2
Extracellular region part	19	5.1	0.00	1.9
Extracellular space	12	3.2	0.02	16.9
**Molecular Function**				
DNA binding	29	7.8	0.05	46.9
Transcription regulator activity	22	5.9	0.04	40.5
Calcium ion binding	18	4.8	0.03	32.1
Transcription factor activity	17	4.6	0.02	19.4
Enzyme binding	10	2.7	0.01	7.7

^a^ Only those GO terms which contained at least 10 differentially expressed genes are listed.

^b^ False discovery rate (x 100).

For genes upregulated in TE, 12 GO terms were listed in the Biological Process group, 12 in the Cell Component group, and 9 in the Molecular Function group (Table 2). GO terms enriched for TE were distinct from those for ICM. A large number of genes represented by GO terms related with metabolism were upregulated in TE including proteolysis (27 genes), oxidation reduction (23 genes), lipid biosynthetic processing (11 genes), steroid metabolic process (10 genes), and peptidase activity (acting on L-amino acid peptides) (22 genes) as well as genes involved in binding reactions [ion binding (86 genes), cation binding (83 genes), metal ion binding (81 genes), calcium ion binding (34 genes) and iron ion binding (12 genes)]. There was also enrichment for genes associated with endo- or exocytosis, membrane transport and alterations in cellular architecture as indicated by GO terms for vesicle-mediated transport (15 genes), actin filament-based process (14 genes), actin cytoskeleton organization (13 genes), cytoskeleton organization (13 genes), plasma membrane (43 genes), endoplasmic reticulum (32 genes), cytoplasmic vesicle (14 genes), vesicle (14 genes), actin cytoskeleton (13 genes), cell projection (12 genes), vacuole (11 genes), endoplasmic reticulum part (11 genes), apical part of cell (10 genes), and cytoskeletal arrangement (20 genes).

**Table 2:** **GO terms enriched for genes upregulated in the TE as compared to ICM**^**a**^

**GO term**	**Count**	**Percent**	**P value**	**FDR**^**b**^
**Biological Process**				
Proteolysis	27	6.4	0.00	6.26
Oxidation reduction	23	5.4	0.01	10.40
Intracellular signaling cascade	20	4.7	0.03	43.10
Ion transport	20	4.7	0.04	50.64
Vesicle-mediated transport	15	3.5	0.00	5.68
Regulation of cell proliferation	15	3.5	0.01	11.09
Actin filament-based process	14	3.3	0.00	0.00
Actin cytoskeleton organization	13	3.1	0.00	0.00
Cytoskeleton organization	13	3.1	0.00	1.22
Lipid biosynthetic process	11	2.6	0.01	12.45
Steroid metabolic process	10	2.4	0.00	0.31
Negative regulation of cell proliferation	10	2.4	0.00	2.24
**Cell Component**				
Plasma membrane	43	10.1	0.04	40.40
Endoplasmic reticulum	32	7.6	0.00	0.00
Cell fraction	16	3.8	0.00	2.50
Cytoplasmic vesicle	14	3.3	0.03	31.04
Vesicle	14	3.3	0.04	36.80
Actin cytoskeleton	13	3.1	0.00	0.04
Membrane fraction	13	3.1	0.01	11.72
Insoluble fraction	13	3.1	0.01	15.08
Cell projection	12	2.8	0.04	41.18
Vacuole	11	2.6	0.00	0.87
Endoplasmic reticulum part	11	2.6	0.00	4.14
Apical part of cell	10	2.4	0.00	0.04
**Molecular Function**				
Ion binding	86	20.3	0.00	0.14
Cation binding	83	19.6	0.00	0.49
Metal ion binding	81	19.1	0.00	0.94
Calcium ion binding	34	8.0	0.00	0.00
Peptidase activity, acting on L-amino acid peptides	22	5.2	0.00	1.41
Cytoskeletal protein binding	20	4.7	0.00	0.00
Actin binding	14	3.3	0.00	0.04
Iron ion binding	12	2.8	0.03	31.90
Lipid binding	11	2.6	0.03	38.82

^a^ Only those GO terms which contained at least 10 differentially expressed genes are listed.

^b^ False discovery rate (x 100).

Functions of differentially expressed genes were further annotated using KEGG (http://www.genome.jp/kegg/). Genes upregulated in ICM were enriched in eight terms (Table 3A). These included pathways involved in lineage commitment (e.g., hematopoietic cell lineage) and differentiation (axon guidance) as well as those involved in maintenance of stemness and self renewal (e.g., pathway in cancer and Jak-STAT signaling pathway). Genes upregulated in TE were enriched in 12 terms (Table 3B). None of the terms were in common with KEGG terms enriched for genes upregulated for ICM. Terms were preferentially related to transmembrane transport (lysosome, aldosterone-regulated sodium resabsorption, and ABC transporters), lipid or steroid metabolism (PPAR signaling pathway, terpenoid backbone biosynthesis, sphingolipid metabolism, steroid hormone biosynthesis, fatty acid metabolism) and other metabolic processes (pantothenate and CoA biosynthesis). Additional file 3 represents a KEGG metabolic pathway map in which pathways that were differentially enriched between ICM and TE were identified using iPath2.0 (http://pathways.embl.de/). Note the increased metabolic activity in TE as compared to ICM.

**Table 3:** KEGG Pathways enriched for genes upregulated in the inner cell mass or trophectoderm

**Term**	**Genes**
Upregulated in Inner Cell Mass (A)	
Antigen processing and presentation	*CD74*, *CD8B*, *HSPA1L*, *HSPA6*, *PSME1*, *BoLA*-*DRB3*
Complement and coagulation cascades	*A2M*, *F2R*, *C1R*, *PLAUR*, *C4BPA*,
Chemokine signaling pathway	*ITK*, *CCL24*, *CXCL7*, *GNAI1*, *GNB5*, *GNG7*, *PLCB1*, *STAT1*, *STAT4*, *STAT3*
Axon guidance	*EPHA4*, *CHP*, *DPYSL2*, *GNAI1*, *ROBO1*, *SEMA4G*, *SLIT2*
Arrhythmogenic right ventricular cardiomyopathy (ARVC)	*CDH2*, *DES*, *GJA1*, *ITGA2*, *TCF7L2*
Pathways in cancer	*CDKN2B*, *FGF12*, *FGF16*, *ITGA2 MMP9*, *PDGFRA*, *STAT1*, *STAT4*, *STAT3*, *TCF7L2*, *FOS*, *KIT*,*WNT*
Jak-STAT signaling pathway	*IL12RB2*, *IL19*, *IL6ST*, *IL7*, *STA1*, *STAT4*, *STAT3*, *SPRY2*
Hematopoietic cell lineage	*CD1A*, *CD8B*, *ITGA2*, *IL7*, *KIT*
Upregulated in Trophectoderm (B)	
Lysosome	*ATP6V0A4*, *GM2A*, *NPC*, *CTSB*, *CTSH*, *CTSL2*, *CTNS*, *GLAA*, *GALC*, *MANBA*, *PLA2G15*, *SCARB2*, *ATP6V0C*, *SLC11A2*
Steroid biosynthesis	*NSDHL*, *CYP41A1*, *FDFT1*, *SC4MOL*
Aldosterone-regulated sodium reabsorption	*ATP1B3*, *NEDD4L*, *PRKCG*, *SGK1*, *SFN*
Vascular smooth muscle contraction	*ACTA2*, *ACTG2*, *CALD1*, *CALML5*, *ITPR2*, *MYLK*, *MYL6*, *PRKCH*, *PRKCG*
PPAR signaling pathway	*ACSL4*, *AXSL6*, *FABP5*, *ACSL3*, *SCD*, *SCP2*
Phosphatidylinositol signaling system	*CALML3*, *ITPR2INPP4B*, *INPP5D*, *PRKCG*, *SYNJ1*
Pantothenate and CoA biosynthesis	*BCAT1*, *ENPP1*, *ENPP3*
Terpenoid backbone biosynthesis	*HMGCR*, *ACAT2*, *IDI1*
Sphingolipid metabolism	*UGCG*, *GLA*, *GALC*, *SGPP1*
Steroid hormone biosynthesis	*UGT1A1*, *UGT1A6*, *CYP11A1*, *CYP3A28*, *HSD3B1*
Fatty acid metabolism	*ACAT2*, *ACSL4*, *ACSL6*, *ACSL3*
ABC transporters	*ABCA3*,*ABCB1*, *ABCC2*, *ABCG5*

### K-mean clustering

The 870 genes that were differentially expressed between ICM and TE were clustered into 8 clusters, with 2, 4, 7, 9, 23,48, 149 and 628 genes in each cluster (Additional file 4). The biggest cluster (628 genes) contained 72.2% of all the significant genes and genes were included from almost all the overrepresented pathways (Table 3). Therefore, the k-mean analysis did not disclose much information on functional expression patterns of differentially expressed genes.

### Comparison of ICM-TE differences in the bovine with the mouse and human

The literature was used to identify a group of genes that have been identified as being expressed by ICM, TE or embryonic stem cells in the mouse [29-32] or human [33-38] (Additional file 5). Among the 119 genes considered characteristic of ICM or embryonic stem cells, 8 were significantly upregulated in ICM (*KDM2B*, *NANOG*, *SOX2*, *SPIC*, *STAT3*, *ZX3HAV1*, and *OTX2*) and two (*IL6R* and *TFRC*) tended (P=0.06 or less) to be upregulated in ICM. Conversely, 6 genes considered as being expressed in ICM or embryonic stem cells in the mouse or human were upregulated in the TE (*DAB2*, *DSP*, *GM2A*, *SCD*, *SSFA2*, and *VAV3*). Of 49 genes considered characteristic of TE, 12 (*AQP11*, *ATP1B3*, *CGN*, *CYP11A*, *DSC2*, *ELF5*, *GATA3*, *HSD3B1*, *KRT18*, *MSX2*, *SFXN or TJP2*) were upregulated in TE. *CDH24*, a cadherin reported to be upregulated in the TE of the human [33], was expressed in higher amounts in the ICM.

We also examined expression of ruminant-specific genes known to be upregulated in TE. The three examined, *IFNT1*[39], *PAG2*[40], and *TKDP1*[41], were upregulated in TE.

We evaluated differences in expression between ICM and TE for genes that have been shown in the mouse [7] to be important for segregation of ICM and TE lineages and subsequent TE differentiation (Table 4). Expression of two genes important for ICM commitment, *NANOG* and *SOX2*, was significantly higher for ICM than TE while expression of two other genes important for ICM commitment, *POU5F1* and *SALL4*, did not differ significantly between ICM and TE. Numerically, expression of these latter two genes was higher for ICM. Four genes were examined that are important for TE commitment – *CDX2*, *GATA3*, *TEAD4*, and *YAP1*. Expression of *GATA3* was significantly higher for TE but there were no significant differences in expression between ICM and TE for the other three genes. One gene important for differentiation of TE later in development, *ELF5*, was expressed in higher amounts in TE (adjusted P=0.022) whereas another, *EOMES*, was barely detectable and not different between ICM and TE.

**Table 4:** **Differences in expression between ICM and TE for genes involved in segregation of ICM and TE in mice**^**a**^

**Gene symbol**	**Role in mouse**	**Mean counts**, **ICM**	**Mean count**, **TE**	**Fold change**, **TE**/**ICM**	**Adjusted P value**
*CDX2*	TE commitment	5.7	2.8	0.49	0.780
*ELF5*	TE differentiation	5.3	28.9	5.41	0.022
*GATA3*	TE commitment	363.6	976.7	2.69	0.018
*EOMES*	TE differentiation	1.4	0.2	0.16	0.934
*NANOG*	ICM commitment	3014.8	620.9	0.21	0.000
*POU5F1*	ICM commitment	2394.1	1873.5	0.78	0.605
*SALL4*	ICM commitment	5.3	3.8	0.71	0.893
*SOX2*	ICM commitment	816.2	360.7	0.44	0.005
*TEAD4*	TE commitment	7.1	12.0	1.69	0.894
*YAP1*	TE commitment	47.9	43.0	0.90	1.000

^a^ Source: Chen et al. [7].

### Characteristics of promoter regions of genes differentially expressed between ICM and TE

The region spanning nucleotide sequences located 200 bp upstream to 50 bp downstream of the transcription start site was examined for presence of putative TFBS for each gene that was differentially expressed between ICM and TE. Binding sites for three transcription factors (PLAG1, RELA and RREB1) were significantly enriched for genes overexpressed in the ICM while binding sites for nine transcription factors (EGR1, GABPA, KLF4, MYF5, SP1, MZF1, NHLH1, PAX5 and ZFX) were significantly enriched for TE. For 11 of 12 transcription factors identified as being used to regulate genes overexpressed in ICM or TE, there was no difference in expression level between ICM and TE. The exception was for *EGR1*, where expression was upregulated in ICM (Additional file 2), even though the TFBS was enriched for genes overexpressed in TE.

### Differences in promoter CpG islands between genes overexpressed in ICM or TE

The percent of genes overexpressed in ICM that were classified as CpG positive (46.6%) was lower (P<0.05) than for genes overexpressed in TE (55.3%). Moreover, the percent of genes classified as CpG positive for genes overexpressed in either tissue was higher than the percent that were classified as CpG positive for the entire bovine genome (39.4%). Thus, DNA methylation may play a greater role for regulation of genes differentially regulated in the ICM and TE than it does for the genome as a whole.

Of the genes that were differentially regulated for ICM and TE, three were genes involved in epigenetic modification. These were *DNMT1* and *KDM2B*, overexpressed in ICM, and *DNMT3A* like sequence, overexpressed in TE (Additional file 2).

### Confirmation of differences in gene expression between ICM and TE by quantitative PCR

**Figure 1:**
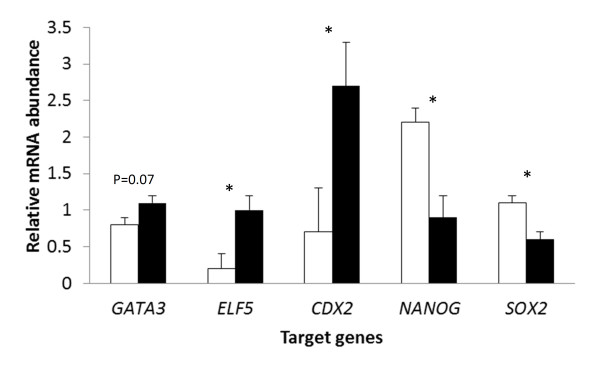
**Differences between inner cell mass (ICM) and trophectoderm (TE) in expression of 6 select genes as determined by quantitative PCR.** Blastocysts were harvested at Day 7 and ICM and TE separated by magnetic activated cell sorting. Data represent least-squares means ± SEM of results from six biological replicates. Open bars represent ICM and filled bars TE. *=P<0.05.

Using isolated ICM and TE from a separate set of blastocysts than used for SOLiD sequencing, qPCR was performed to verify treatment effects on gene expression for 6 genes (*GATA3*, *ELF5*, *CDX2*, *NANOG* and *SOX2*). Results for differences between ICM and TE were generally consistent with results from deep sequencing (Figure 1). In particular, expression was higher for TE than ICM for *GATA3* (P=0.07) and *ELF5* (P<0.05) and was higher for ICM than TE for *NANOG* (P<0.05) and *SOX2* (P<0.05). One discrepancy with deep sequencing results was for *CDX2*. While there was no significant difference between ICM and TE in the deep sequencing data base (Table 4), mRNA for *CDX2* was higher for TE than ICM as determined by qPCR (Figure 1).

## Discussion

Differentiation in the mammalian embryo is dependent upon spatial position - cells on the inside of the embryo remain pluripotent for a period until initiation of gastrulation while cells on the outer face of the embryo differentiate into TE and ultimately form much of the extraembryonic membranes. Here, using magnetic-assisted cell sorting and high-throughput next generation sequencing, we show the consequences of spatial differences between ICM and TE and subsequent divergence in lineage commitment for expression of genes regulating pluripotency and lineage commitment, cellular metabolism, and interactions with the maternal system.

Commitment towards the ICM lineage in the mouse is maintained by actions of Pou5f1 (Oct4), Sall4, Sox2 and Nanog; Cdx2 in the TE inhibits *Pou5f1* expression and allows differentiation of extraembryonic membranes [3,4,7]. In the bovine, too, *SOX2* and *NANOG* were overexpressed in ICM but expression of *POU5F1* and *SALL4* were not significantly different between ICM and TE. A high degree of expression of *POU5F1* in the TE was expected because differences in the regulatory region of the *POU5F1* gene in cattle as compared to the mouse gene make *POU5F1* resistant to regulation by CDX2 [6]. Nonetheless, *POU5F1* expression is greater in the ICM of cattle [6,42]. In the present study, expression of both *POU5F1* and *SALL4* were numerically greater for ICM; failure to find significant differences between ICM and TE may represent the small sample size. It should also be kept in mind that embryos produced in vitro have altered patterns of gene expression relative to embryos produced in vivo [43]. Such alterations could change some of the differential gene expression between ICM and TE, as has been reported for the mouse embryo [44].

Analysis of genes upregulated in ICM provides some clues as to the signaling pathways required for specification, pluripotency, and other functions of the ICM. A total of 8 genes in the KEGG Jak-STAT signaling pathway were upregulated. In mice, LIF, which signals through the Jak-STAT pathway, can promote pluripotency of cells derived from the ICM [45]. While LIF cannot cause bovine ICM cells to develop into stem cells [46], other molecules that signal through the Jak-STAT pathway are likely to be involved in regulation of the ICM. Several genes related to cellular migration were upregulated in ICM, as indicated by enrichment of the chemokine signaling pathway (10 genes) and axon guidance (7 genes) GO terms. In the mouse, blastomeres of the ICM can change position, at least in part to align position with subsequent formation of primitive endoderm [47-49]. Perhaps, movement is directed by guidance molecules such as chemokines.

Outer cells of the mouse blastocyst are committed towards the TE lineage through the actions of *Yap1*, *Tead4*, *Gata3*, and *Cdx2* ([3,4,7]. We found no difference in *CDX2* expression between ICM and TE using deep sequencing even though it is well established that the gene is expressed to a greater extent in TE of the bovine [6,9,42] and *CDX2* expression was higher in TE than ICM in the qPCR experiment. *CDX2* expression was very low in the deep sequencing experiment, especially compared to that of *POU5F1*. One possibility is that differences in *CDX2* expression between TE and ICM at Day 7 (as detected by qPCR) become reduced at Day 8. Like seen earlier [6], other homologues of *CDX2* were not detected (*CDX1*) or were nearly non-detectable (*CDX4*) (Additional file 2).

Another gene involved in TE lineage, *GATA3*, was expressed in higher amounts in TE. A similar but non-significant difference in expression between ICM and TE was noted earlier [42]. There was no significant difference in *TEAD4* or *YAP1* expression between ICM and TE. Similar findings were observed in the bovine for *TEAD4*[42]. A gene involved in development of extraembryonic ectoderm in mice, *ELF5*[7], was overexpressed in TE whereas another gene involved in development of extraembryonic membranes, *EOMES*, was barely detectable. In fact, there appears to be an absence or very low expression of *EOMES* in TE between day 7 and 15 of gestation in cattle [6]. In addition, by Day 11 of gestation, trophoblast expression of *ELF5* is inhibited and becomes limited to the epiblast [50].

It is notable that several genes characteristically expressed in ICM of mouse or human, *DAB2*, *DSP*, *GM2A*, *SCD*, *SSFA2*, and *VAV3*, [30,32,37] were significantly overexpressed in the TE of the bovine while *CDH24*, reported to be upregulated in the TE of the human [33], was expressed in higher amounts in the ICM of the bovine. *Dsp* and *Dab2* are indispensible for embryonic development in mice and homologous recombination causes postimplantation embryonic failure [51,52]. Clearly, as first shown by Berg et al. [6], divergent evolution in the control of early embryonic development means that study across a wide array of species is required to understand developmental processes fully.

By virtue of its position in the embryo, polarized morphology [53] and tight junctions between its member cells [1], the TE is fated to be the cell lineage through which the blastocyst interacts directly with the mother in terms of nutrient exchange, maternal-conceptus communication, and placentation. It appears that executing these functions places increased metabolic demands on the TE as compared to the ICM as indicated by upregulation of genes involved in metabolism, particularly those involved in lipid metabolism. Lipid accumulation in cultured bovine embryos is greater for TE than ICM, although the difference depends upon medium [54,55].

It is through the TE that nutrients enter the embryo and from the TE that secretory products of the embryo must enter the uterine environment. Consistent with a role for the TE in uptake and delivery was upregulation of genes involved in endo- or exocytosis and membrane transport. Lysosomal-like structures have been reported to be more abundant in TE than ICM in cattle, at least for certain media [54,55], and the mouse [53].

Molecules involved in signaling to the mother that were upregulated in TE include *IFNT1*, *PAG2* and *TKDP1*. The role for *IFNT1* is to act on the maternal endometrium to block luteolytic release of prostaglandin F_2α_[39,56]. While this action is initiated later in pregnancy, between Day 15 and 17 of gestation, secretion of IFNT occurs as early as the blastocyst stage [57]. *TKDP1* is a member of the Kunitz family of serine proteinase inhibitors and may function to limit trophoblast invasiveness in species like the cow with epitheliochorial placentation [41]. Little is known about the role of *PAG2*, which is the mostly abundantly expressed of at least 22 transcribed *PAG* genes [40]. Unlike some *PAG* genes (the so-called “modern” clade), whose expression is limited to trophoblast giant cells formed later in development, *PAG2* is expressed widely in the cotyledonary trophoblast and is predicted to be an active aspartic proteinase [58].

*IFNT1*, *PAG2* and *TKDP1* are all genes that are phylogenetically-restricted to ruminants. Another conceptus product that is produced more widely in mammals is estrogen. The role for embryonic estrogen is not known for most species but blastocyst estrogen has been suggested to be involved in hatching from the zona pellucida in hamsters [59] and in conceptus growth in the pig [60]. The bovine blastocyst, too, produces estrogen [61] and the upregulation of genes involved in terpenoid backbone biosynthesis and steroid hormone biosynthesis suggest that the primary source of blastocyst estrogens is the TE.

Following blastocyst formation, the ruminant trophoblast undergoes a series of developmental steps that are dependent on changes in cell shape and spatial position, including hatching (which requires actin-based trophectodermal projections [59]), elongation (which leads to an increase in size of the conceptus from about 0.16 mm at Day 8 to as much as 100 mm or more at Day 16 [62]) and eventual attachment to the maternal endometrium (commencing around Day 20 in the cow [63]. The upregulation of genes in the trophoblast for ontologies such as actin filament-based process, actin cytoskeleton organization, cell projection and cytoskeletal arrangement reflects the extensive changes in cell architecture required for these processes. In addition, three cathepsin genes, *CTSB*, *CTSH* and *CTSL2*, were upregulated in TE; these proteinases have been implicated in blastocyst hatching [59,64].

Differences in gene expression between ICM and TE are probably due in large part to differences in transcription factor usage and to epigenetic modifications. Binding sites for the transcription factors PLAG1, RELA and RREB1 were enriched for genes overexpressed in ICM while binding sites for nine transcription factors (EGR1, GABPA, KLF4, MYF, SP1, MZF1, NHLH1, PAX5 and ZFX) were significantly enriched for TE. RELA is a subunit for NFκB, which in turn has been implicated in differentiation of trophoblast lineages from embryonic stem cells [65] and in function of trophoblast giant cells [66]. Several of the transcription factors associated with genes upregulated in TE are involved in hematopoiesis, including EGR1 [67], GABPA [68], MZF1 [69], and ZFX [70]. One of these transcriptional factors, GABPA, can enhance *Pou5f1* expression in mouse embryonic stem cells [71] and another, KLF4, is a key regulator of maintenance and induction of pluripotency [72]. The overall picture is one where hematopoiesis and stemness is under positive regulation in the TE. Another transcription factor associated with regulation of genes upregulated in TE was SP1. This protein exerts several actions to regulate trophoblast development and function, including activation of expression of other transcription factors such as *Tfap2c*[73] and *Id1*[74]. In the cow, SP1 becomes limited to binucleate cells of the trophoblast by Day 25 [75].

DNA methylation could be important for regulation of gene expression in the blastocyst because the promoter regions of over half of the genes that were upregulated in ICM or TE were classified as CpG positive. Indeed, the percent of genes classified as CpG positive for genes overexpressed in ICM or TE was higher than the percent that were classified as CpG positive for the entire bovine genome. Slightly fewer genes that were overexpressed in ICM were classified as CpG-positive than for genes that were overexpressed in TE, which might suggest more inhibition of gene expression by methylation in TE. It is noteworthy, however, that Niemann et al. [76] did not find a correlation between degree of CpG island methylation and amount of embryonic expression for eight genes examined. Recent evidence has been interpreted to signify that it is not the methylation state of individual CpG that determine gene expression but rather the methylation status of large regions of DNA that span multiple genes [77].

In cattle, there are conflicting data as to whether DNA methylation is less extensive for ICM or for TE in both embryos produced in vitro and by somatic cell nuclear transfer [78-80], Another epigenetic mark, H3K27me3, is similar for both cell types [81]. Of the genes that were differentially regulated for ICM and TE, three were genes involved in epigenetic modification. Two were overexpressed in ICM: *DNMT1*, involved in maintenance of DNA methylation during succeeding cell divisions [77], and *KDM2B*, a lysine-specific histone dimethylase which catalyzes demethylation of H3K4 and H3K6 [82,83]. In contrast, a *DNMT3A* like sequence, which establishes DNA methylation during development and also participates in methylation maintenance [77], was overexpressed in TE. The presence of increased transcript abundance for *DNMT3A* could be interpreted to mean that *de novo* DNA methylation occurs to a greater degree in TE, as is indicated by studies with embryos produced in vitro [79] and by somatic cell nuclear cloning [80]. Further research is necessary to determine differences in DNA methylation between TE and ICM at the gene-specific and genome-wide level.

In general, analysis of a separate set of isolated ICM and TE by qPCR confirmed the results obtained for differences between cell types by deep sequencing. The exception was for *CDX2*, where there was no difference in expression as determined by SOLiD sequencing but where expression was greater for TE than ICM as determined by qPCR. The discrepancy could reflect either day of sampling differences (as discussed earlier) or, given the often-repeated observation that *CDX2* is expressed to a greater extent in TE than ICM [6,9,42], an error induced by the deep sequencing procedure.

In conclusion, differentiation of blastomeres of the morula-stage embryo into the ICM and TE of the blastocyst is accompanied by differences between the two cell lineages in expression of genes controlling metabolic processes, endocytosis, hatching from the zona pellucida, paracrine and endocrine signaling with the mother, and genes supporting the changes in cellular architecture, stemness, and hematopoiesis necessary for development of the trophoblast. Much of the process leading to this first differentiation event seems to be under the control of genes such as *NANOG* and *GATA3* that play central role in lineage commitment in the mouse. As found by others also [6,42], there are fundamental differences from the mouse. Understanding the nature of the process of preimplantation development in mammals will necessarily require a comparative approach based on study of a variety of animal models.

## Conclusions

Analysis of gene expression indicated that differentiation of blastomeres of the morula-stage embryo into the ICM and TE of the blastocyst is accompanied by differences between the two cell lineages in expression of genes controlling metabolic processes, endocytosis, hatching from the zona pellucida, paracrine and endocrine signaling with the mother, and genes supporting the changes in cellular architecture, stemness, and hematopoiesis necessary for development of the trophoblast.

## Abbreviations

DAVID: Database for annotation, visualization and integrated discovery; GO: Gene ontology; KEGG: Kyoto encyclopedia of genes and genomes; ICM: Inner cell mass; SSE: Sum of squares error; TFBS: Transcription factor binding sites; TE: Trophectoderm.

## Competing interests

The authors have declared that no competing interests exist.

## Author contributions

Conceived and designed the experiments: MO, PJH WGF. Performed the experiments: MO, SS, KBD, MJS. Analyzed the data: MO, MS, J-QY, FY, RY, SW, KN, PJH. Wrote initial drafts of the paper: MO PJH. All authors read and approved the final manuscript.

## Supplementary Material

Additional file 1 Formulas used for enrichment analysis for transcription factor binding sites. Click here for file

Additional file 2 **Differences in gene expression between ICM and TE.** Genes in which the adjusted P value was <0.05 are color coded (blue are upregulated in ICM and red are upregulated in TE). Click here for file

Additional file 3 KEGG metabolic pathway map in which pathways that were differentially enriched between ICM (blue) and TE (red) were identified using iPath2.0. Click here for file

Additional file 4 **Heatmap constructed by k-mean clustering of the 870 genes that differ in expression between ICM and TE.** The colors in the map display the relative standing of the reads count data; blue indicates a count value that is lower than the mean value of the row while red indicates higher than the mean. The shades of the color indicate how far away the data from the mean value of the row. Columns represent individual samples of ICM (IC) and TE (TC). Click here for file

Additional file 5 Differences in expression between inner cell mass (ICM) and trophectoderm (TE) for genes considered as being characteristically expressed by ICM and TE in human or mouse. Click here for file
