# Supplementary material for: Global gene expression of the inner cell mass and trophectoderm of the bovine blastocyst
Source: BMC Dev Biol. 2012 Nov 6;12:33. doi: 10.1186/1471-213X-12-33 (PMC3514149; doi:10.1186/1471-213X-12-33)
Supplement: Additional file 1 — Formulas used for enrichment analysis for transcription factor binding sites. [file 1471-213X-12-33-S1.pdf]

Formula 1: Position-specific weight matrix (PSWM) scores

$$score = \sum_{i=1}^{l} \log\left(\left(n_{if} + 1\right) / (N_i + 4) * (1/4)\right)$$

where l: length of the matrix; n_{if}: the observed number of base 'f' (A, C, G or T) at the i-th position in the training; N_i: sum of the observed number of all bases at i-th position.

Formula 2: Ratio of the score to the maximum score

$$ratio = \frac{score - \min(score)}{\max(score) - \min(score)}$$

where min(*score*): minimum value of each PSWM, max(*score*): maximum value of each PSWM.

Formula 3: Hypergeometric distribution

$$\sum_{x=i}^{Np} \frac{\binom{Np}{x} \bullet \binom{N(1-p)}{n-x}}{\binom{N}{x}}$$

where N: total number of promoters, p: expected frequency of the promoters associated with a given PSWM (number of associated promoters/N), n: total number of promoters belonging to a dataset of concern , and i : number of the promoters associated with a given PSWM in a dataset.
