# Supplementary material for: Global gene expression of the inner cell mass and trophectoderm of the bovine blastocyst
Source: BMC Dev Biol. 2012 Nov 6;12:33. doi: 10.1186/1471-213X-12-33 (PMC3514149; doi:10.1186/1471-213X-12-33)
Supplement: Additional file 3 — KEGG metabolic pathway map in which pathways that were differentially enriched between ICM (blue) and TE (red) were identified using iPath2.0. [file 1471-213X-12-33-S3.pdf]

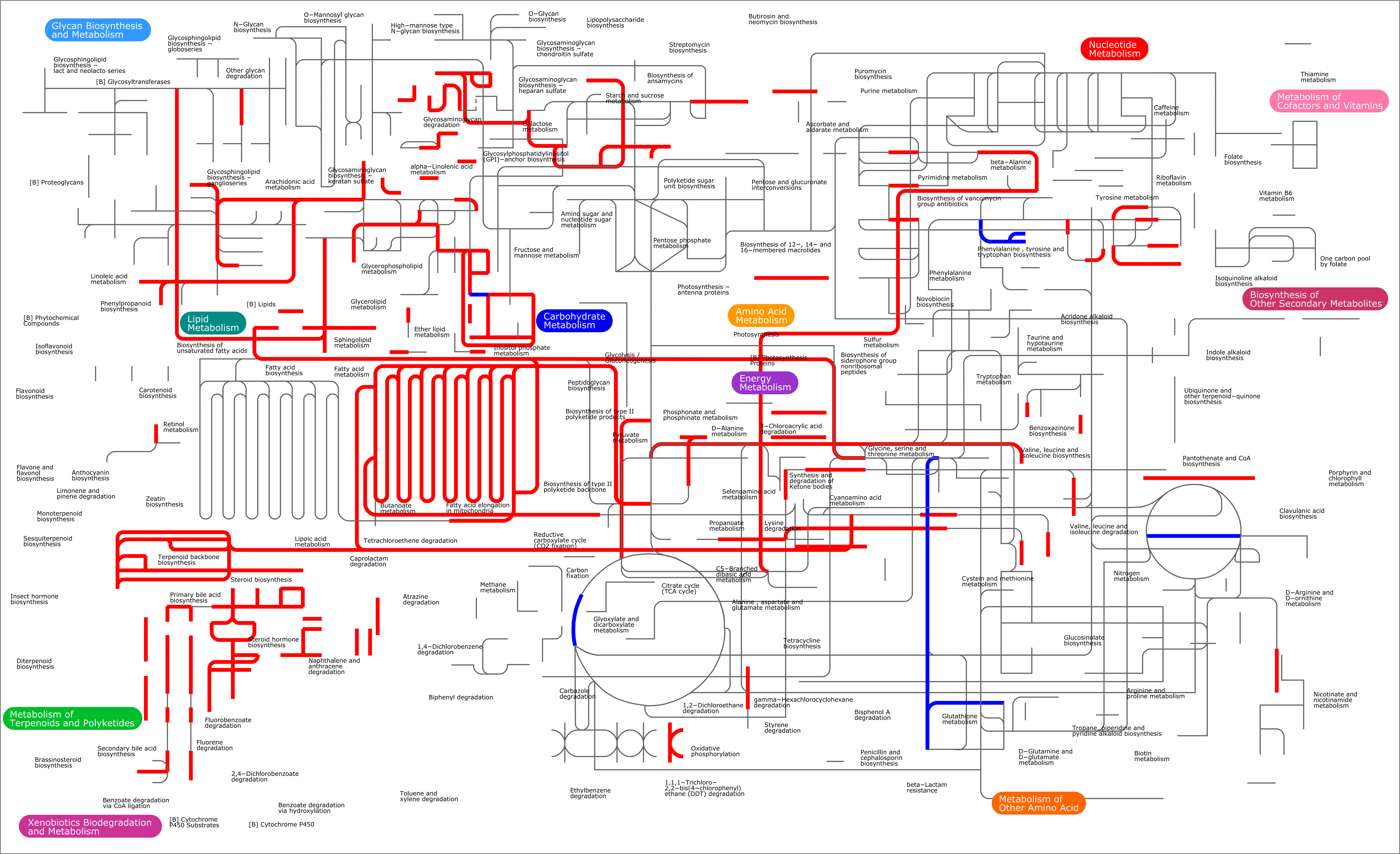

Glycan Biosynthesis and Metabolism
Glycosphingolipid biosynthesis − lact and neolacto series
Glycosphingolipid biosynthesis − globoseries
N−Glycan biosynthesis
O−Mannosyl glycan biosynthesis
High−mannose type N−glycan biosynthesis
O−Glycan biosynthesis
Lipopolysaccharide biosynthesis
Butirosin and neomycin biosynthesis
Nucleotide Metabolism
Other glycan degradation
[B] Glycosyltransferases
Glycosaminoglycan biosynthesis − chondroitin sulfate
Streptomycin biosynthesis
Glycosaminoglycan biosynthesis − heparan sulfate
Biosynthesis of ansamycins
Puromycin biosynthesis
Purine metabolism
Thiamine metabolism
Metabolism of Cofactors and Vitamins
Starch and sucrose metabolism
Glycosaminoglycan degradation
Galactose metabolism
Caffeine metabolism
Ascorbate and aldarate metabolism
Folate biosynthesis
Glycosylphosphatidylinositol (GPI)−anchor biosynthesis
alpha−Linolenic acid metabolism
beta−Alanine metabolism
Riboflavin metabolism
[B] Proteoglycans
Glycosphingolipid biosynthesis − ganglioseries
Arachidonic acid metabolism
Glycosaminoglycan biosynthesis − keratan sulfate
Polyketide sugar unit biosynthesis
Pentose and glucuronate interconversions
Pyrimidine metabolism
Vitamin B6 metabolism
Biosynthesis of vancomycin group antibiotics
Tyrosine metabolism
Amino sugar and nucleotide sugar metabolism
Pentose phosphate metabolism
Biosynthesis of 12−, 14− and 16−membered macrolides
Phenylalanine , tyrosine and tryptophan biosynthesis
One carbon pool by folate
Fructose and mannose metabolism
Glycerophospholipid metabolism
Linoleic acid metabolism
Phenylalanine metabolism
Isoquinoline alkaloid biosynthesis
Photosynthesis − antenna proteins
Phenylpropanoid biosynthesis
[B] Lipids
Glycerolipid metabolism
Novobiocin biosynthesis
Biosynthesis of Other Secondary Metabolites
[B] Phytochemical Compounds
Lipid Metabolism
Carbohydrate Metabolism
Amino Acid Metabolism
Acridone alkaloid biosynthesis
Ether lipid metabolism
Photosynthesis
Sulfur metabolism
Taurine and hypotaurine metabolism
Isoflavonoid biosynthesis
Biosynthesis of unsaturated fatty acids
Sphingolipid metabolism
Inositol phosphate metabolism
Glycolysis / Gluconeogenesis
[B] Photosynthesis Proteins
Biosynthesis of siderophore group nonribosomal peptides
Indole alkaloid biosynthesis
Fatty acid biosynthesis
Fatty acid metabolism
Energy Metabolism
Tryptophan metabolism
Flavonoid biosynthesis
Carotenoid biosynthesis
Peptidoglycan biosynthesis
Ubiquinone and other terpenoid−quinone biosynthesis
Retinol metabolism
Biosynthesis of type II polyketide products
Phosphonate and phosphinate metabolism
D−Alanine metabolism
3−Chloroacrylic acid degradation
Benzoxazinone biosynthesis
Pyruvate metabolism
Glycine, serine and threonine metabolism
Valine, leucine and isoleucine biosynthesis
Pantothenate and CoA biosynthesis
Flavone and flavonol biosynthesis
Anthocyanin biosynthesis
Synthesis and degradation of Ketone bodies
Porphyrin and chlorophyll metabolism
Limonene and pinene degradation
Biosynthesis of type II polyketide backbone
Selenoamino acid metabolism
Zeatin biosynthesis
Cyanoamino acid metabolism
Butanoate metabolism
Fatty acid elongation in mitochondria
Monoterpenoid biosynthesis
Clavulanic acid biosynthesis
Propanoate metabolism
Lysine degradation
Valine, leucine and isoleucine degradation
Sesquiterpenoid biosynthesis
Lipoic acid metabolism
Tetrachloroethene degradation
Reductive carboxylate cycle (CO2 fixation)
Terpenoid backbone biosynthesis
Caprolactam degradation
Carbon fixation
C5−Branched dibasic acid metabolism
Nitrogen metabolism
Steroid biosynthesis
Methane metabolism
Citrate cycle (TCA cycle)
Cystein and methionine metabolism
Insect hormone biosynthesis
Primary bile acid biosynthesis
Atrazine degradation
Alanine , aspartate and glutamate metabolism
D−Arginine and D−ornithine metabolism
Glyoxylate and dicarboxylate metabolism
Steroid hormone biosynthesis
Tetracycline biosynthesis
Glucosinolate biosynthesis
1,4−Dichlorobenzene degradation
Diterpenoid biosynthesis
Naphthalene and anthracene degradation
Biphenyl degradation
Carbazole degradation
Arginine and proline metabolism
gamma−Hexachlorocyclohexane degradation
1,2−Dichloroethane degradation
Bisphenol A degradation
Nicotinate and nicotinamide metabolism
Glutathione metabolism
Metabolism of Terpenoids and Polyketides
Fluorobenzoate degradation
Styrene degradation
Tropane, piperidine and pyridine alkaloid biosynthesis
Fluorene degradation
Oxidative phosphorylation
Secondary bile acid biosynthesis
Penicillin and cephalosporin biosynthesis
D−Glutamine and D−glutamate metabolism
Biotin metabolism
Brassinosteroid biosynthesis
2,4−Dichlorobenzoate degradation
1,1,1−Trichloro− 2,2−bis(4−chlorophenyl) ethane (DDT) degradation
Toluene and xylene degradation
Ethylbenzene degradation
beta−Lactam resistance
Benzoate degradation via CoA ligation
Metabolism of Other Amino Acid
Benzoate degradation via hydroxylation
Xenobiotics Biodegradation and Metabolism
[B] Cytochrome P450 Substrates
[B] Cytochrome P450
