# Supplementary figures and images for: Global gene expression of the inner cell mass and trophectoderm of the bovine blastocyst
Source: BMC Dev Biol. 2012 Nov 6;12:33. doi: 10.1186/1471-213X-12-33 (PMC3514149; doi:10.1186/1471-213X-12-33)

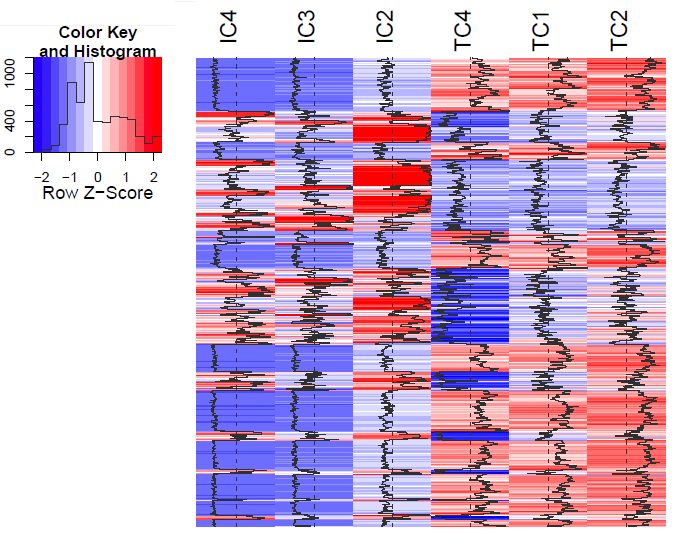

Supplement: Additional file 4 — Heatmap constructed by k-mean clustering of the 870 genes that differ in expression between ICM and TE. The colors in the map display the relative standing of the reads count data; blue indicates a count value that is lower than the mean value of the row while red indicates higher than the mean. The shades of the color indicate how far away the data from the mean value of the row. Columns represent individual samples of ICM (IC) and TE (TC). [file 1471-213X-12-33-S4.png]
