# Supplementary material for: Global gene expression of the inner cell mass and trophectoderm of the bovine blastocyst
Source: BMC Dev Biol. 2012 Nov 6;12:33. doi: 10.1186/1471-213X-12-33 (PMC3514149; doi:10.1186/1471-213X-12-33)
Supplement: Additional file 5 — Differences in expression between inner cell mass (ICM) and trophectoderm (TE) for genes considered as being characteristically expressed by ICM and TE in human or mouse. [file 1471-213X-12-33-S5.pdf]

Additional File 4. Differences in expression between inner cell mass (ICM) and trophectoderm (TE) for genes considered as being characteristically expressed by ICM, embryonic stem cells and TE in human or mouse. [a]

**Genes Considered Characteristic of ICM or embryonic stem cells**												
ABL2	ACOT9	AMD1	ARHGER18	BXL2L14	CANX	CCNB1	CDCA2	CENPF	CLDN7	CYP2S1	**DAB2**	DDX3X
DNMT3L	**DSP**	EI24	EIF1AX	EIF2S1	EIF5B	EPRS	FGFR2	FGFR3	FLG	FOXD3	FOXH1	GATA2
**GATA3**	GATAD1	GJC1	GLTSCR2	**GM2A**	GPD1L	H19	HMGB1	HN1	HSD17B11	IFITM2	IGF2R	IL17RA
**IL6R** [b]	ITGA5	ITGA6	JAK2	JUNB	**KDM2B**	KLF2	KTN1	LAMC1	LAMP2	LEFTY1	LRPAP1	MAP3K3
MORC1	MRPL15	MTCH2	MUC15	MYC	**NANOG**	NANOS1	NODAL	**OTX2**	PAX8	PDPN	PEG3	PEX3
PLK1	POU2F1	POU5F1	PPARD	PTP4A1	PTTG1IP	PUM1	PUM2	PYGB	RAB12	PYGB	RAB12	RAB5C
RAB6A	RELL1	RHPN2	RPL14	RPL19	RPL32	RPL7A	RUNX1	SALL4	SBNO1	**SCD**	SIN3B	SLIT3
SMAD2	SMAD4	SMAD5	SNX3	**SOX2**	SPATA13	SPETA5L1	**SPIC**	SRRM1	**SSFA2**	STAG2	**STAT3**	TACC1
TAL1	TAXBP1	**TFRC** [c]	TGFBR1	TGFBR3	TMED2	TOP2A	TPRKB	TRNAU1AP	**VAV3**	XIST	ZBTB34	**ZC3HAV1**
ZFP42	ZNF296											
**Genes Considered Characteristic of TE**												
**AQP11**	ARNT2	ASCL2	**ATP1B3**	BMP4	CDH19	CDH22	**CDH24**	CDX1	CDX2	CELSR2	CGB	CGB1
CGB2	**CGN** [d]	CLDN10	CLDN2	CSNK1A1	**CYP11A**	DAAM1	**DSC2**	**ELF5**	EOMES	ESRRB	FGFR2	GCM1
HAND1	HLA-G	**HSD3B1**	**IFNT1**	ID2	IRX3	JAM2	KRT1	**KRT18**	LEP	LHCGR	**MSX2**	OCLN
**PAG2**	PCDH1	PCDHB7	PSG3	**SFN**	TBX1	TEAD2	TEAD4	**TKDP1**	**TJP2**			

[a] Genes in blue were upregulated in ICM and genes in red were upregulated in TE. The adjusted P value was <0.05 unless indicated by superscripts

[b] adjusted P=0.05

[c] adjusted P=0.06.

[d] adjusted P=0.09.
